# Effect of age and vaccination on extent and spread of *Chlamydia pneumoniae* infection in C57BL/6 mice

**DOI:** 10.1186/1742-4933-9-11

**Published:** 2012-05-17

**Authors:** Taylor Eddens, Sarah Beaudoin, Amanda Steinberger, C Scott Little, Dawn Shell, Benjamin Wizel, Brian Balin, Kerin L Fresa-Dillon

**Affiliations:** 1 Department of Biology, Washington and Jefferson College, Washington, PA, 15301, USA; 2 Department of Pathology, Microbiology, Immunology, and Forensic Medicine and the Center for Chronic Disorders of Aging, Philadelphia College of Osteopathic Medicine, Philadelphia, PA, 19131, USA; 3 Department of Microbiology and Immunology, Center for Pulmonary and Infectious Disease Control, University of Texas Health Center, Tyler, Texas, 75708, USA; 4 Department of Pathology, Microbiology, and Immunology, the Philadelphia College of Osteopathic Medicine, 4170 City Avenue, Philadelphia, PA, 19131, USA; 5 Current address: Intercell AG, Campus Vienna Biocenter 3, Vienna, 1030, Austria

**Keywords:** *Chlamydia pneumoniae*, Aging, Vaccine

## Abstract

**Background:**

*Chlamydia pneumoniae* is an obligate intracellular respiratory pathogen for humans. Infection by *C. pneumoniae* may be linked etiologically to extra-respiratory diseases of aging, especially atherosclerosis. We have previously shown that age promotes *C. pneumoniae* respiratory infection and extra-respiratory spread in BALB/c mice.

**Findings:**

Aged C57BL/6 mice had a greater propensity to develop chronic and/or progressive respiratory infections following experimental intranasal infection by *Chlamydia pneumoniae* when compared to young counterparts. A heptavalent CTL epitope minigene (CpnCTL7) vaccine conferred equal protection in the lungs of both aged and young mice. This vaccine was partially effective in protecting against *C. pneumoniae* spread to the cardiovascular system of young mice, but failed to provide cardiovascular protection in aged animals.

**Conclusions:**

Our findings suggest that vaccine strategies that target the generation of a *C. pneumoniae*-specific CTL response can protect the respiratory system of both young and aged animals, but may not be adequate to prevent dissemination of *C. pneumoniae* to the cardiovascular system or control replication in those tissues in aged animals.

## Rationale and hypothesis

*Chlamydia pneumoniae* is an important respiratory pathogen in humans [1]. Extra-respiratory spread may be common and with consequence: *C. pneumoniae* or chlamydial DNA has been detected in the coronary arteries of 46% of individuals with atherosclerosis, but rarely in individuals without coronary artery disease [2]. *C. pneumoniae*-specific T cells have also been detected within atheromatous plaques [3].

Infection by *C. pneumoniae* is common in western countries, at least 70% of the total population has been infected by age 65 [4,5]. Pneumonia poses a high risk of morbidity and mortality in aged humans, and vaccines against respiratory pathogens are often less effective in the elderly [reviewed in [6]]. Thus, there is a need for vaccine strategies that prevent both respiratory infection and extra-respiratory spread of *C. pneumoniae* in aged, as well as young individuals.

We have previously established in BALB/c mice tthat aging was associated with impaired resolution of respiratory *C. pneumoniae* infection, more extensive inflammation and consolidation within the infected lung, enhanced spread *of C. pneumoniae* to the cardiovascular system and increased inflammation within the heart [7]. We sought to extend these findings to a genetically disparate strain, C57BL/6, which has been shown to mount a strong Th1-polarized immune response specific for *C. pneumoniae*[8,9]. We also hypothesized that a heptavalent cytotoxic T cell (CTL) epitope DNA minigene vaccine (CpnCTL7) [10], which has been shown to generate epitope-specific CD8^+^ CTL capable of IFN-γ and TNF-α secretion and cytolytic activity against *C. pneumoniae* infected macrophages *in vitro*. and reduce mean respiratory bacterial titers in young, *C. pneumoniae*-infected C57BL/6 mice [10], would be less effective in prevention of respiratory and cardiovascular *C. pneumoniae* infection in aged C57BL/6 mice.

## Methods employed

To test this hypothesis, female C57BL/6 mice (National Institutes of Aging) received three injections of 100 μg of *Cpn*CTL7 or VR1012 plasmid DNA as previously described [10]. Twelve days after the third dose, C57BL/6 mice, at 6 and 20 months of age, were infected by intranasal inoculation with 5.0 x 10^5^ IFU of *C. pneumoniae* (AR-39; ATCC, Rockville MD), in HBSS [7]. Uninfected mice received HBSS alone. Mice were euthanized 14 or 28 days after infection by CO_2_ asphyxiation. Lungs and hearts/ascending aortae were removed, snap frozen in liquid nitrogen and stored at −80°C until assay.

To test for the presence of *Chlamydia* in tissue samples, four-well chamber slides (Lab Tech, Naperville, Ill) were seeded with 1.4 x 10^5^ HEp-2 cells/well and incubated overnight at 37°C in 5% CO_2_. Serial 10-fold dilutions of tissue homogenate prepared as described previously [6] were added to the wells. Negative control wells contained HEp-2 cells in media alone. The slides were centrifuged at 390xg for 30 min (Sorvall Legend RT, Kendro Laboratory Products, Asheville, NC). After 2 h at 37°C, cycloheximide (Sigma Scientific, St. Louis, MO, final concentration = 2 μg/ml) was added and the chamber slides were incubated for an additional 72 h at 37°C. Slides were washed with PBS, fixed with Cytofix/Cytoperm (BD Biosciences, San Diego, CA) for 30 min, and washed again in PBS. Slides were treated with a 1X Perm/Wash solution (BD Biosciences, San Diego, CA) for 15 min and stained with 0.2 μg FITC-conjugated *Chlamydia trachomatis* LPS-specific antibody (Catalog #61-C75, Fitzgerald Industries Intl., Concord, MA) in PBS for 60 min at 37°C. Slides were washed, counterstained with a 1:1,000 dilution of bisBenzamide (1 μg/mL, Sigma Scientific, St. Louis, MO) in PBS, and rinsed again. Slides were mounted in Gel/Mount (Biomeda Corp., Foster City, CA), cover-slipped and stored in the dark. Infected cells were counted at 600x magnification using a Nikon Eclipse E800 microscope. Titers were calculated as described previously [7].

One-way analysis of variance (ANOVA) tests were performed using SPSS.

## The CpnCTL7 vaccine provides protection against respiratory *C. pneumoniae* infection in both young and aged C57BL/6 mice

**Figure 1:**
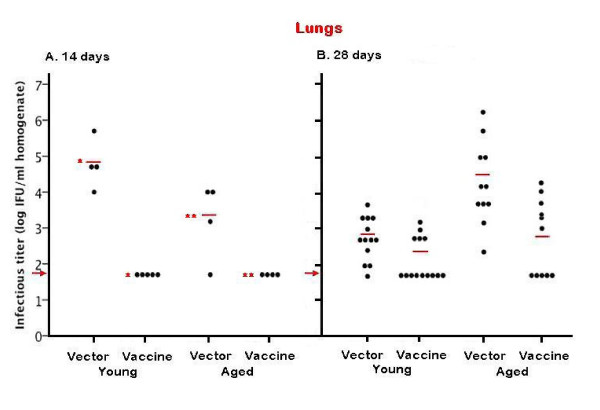
**Recovery of *C. pneumoniae *from the lung 14 and 28 days after intranasal inoculation of 5x10**^**5**^**IFU 6-month-old (young) or 20-month-old (aged) C57BL/6 mice were immunized with 100 μg of *Cpn*CTL7 (vaccine) or VR1012 plasmid DNA (vector) and challenged intranasally with 5x10**^**5**^** IFU *C. pneumoniae *12 days after completion of the immunization protocol.** Mice were euthanized 14 days (**A**) or 28 days (**B**) after infection, the lungs were removed, and lysates prepared as described in Materials and Methods. Viable organisms were recovered and quantified by immunofluorescent staining using FITC-conjugated *Chlamydia*-specific antibody (Fitzgerald 61-C75). The groups of experimentally infected and uninfected age-matched control mice are listed on the X-axis and the number of inclusion forming units/ml of 10 % weight/volume homogenate (log_10_) is displayed on the Y-axis. Each dot represents the concentration (IFU/ml) of *C. pneumoniae* recovered from the organ homogenate of an individual mouse. The red bars indicate the geometric mean of all animals in each group (log_10_) and the red arrow indicates the limit of C. pneumoniae in the detection system. The “**” symbols indicate a statistically significant difference (p = 0.025) between the geometric mean of respiratory titers of aged, vaccinated mice and aged mice receiving vector alone.

At 14 days post-infection (p.i., Figure 1A), all (4/4) non-vaccinated young C57BL/6 mice had evidence of *C. pneumoniae* respiratory infection (mean titer = 6.0 x 10^4^ IFU/ml, range: 1.0 x 10^4^-5.0 x 10^5^ IFU/ml). In contrast, the CpnCTL7 vaccine protected against respiratory *C. pneumoniae* infection in all 5 infected young mice (p = 0.001). Similarly, 3 of 4 (75%) non-vaccinated aged mice had detectable *C. pneumoniae* in the lung; but none of the 4 aged, vaccinated mice had detectable *C. pneumoniae* titers at 14 days p.i. (p = 0.012). These results suggest that the cytolytic T cell response induced by the CpnCTL7 vaccine was of sufficient magnitude in aged mice to provide at least temporary protection against *C. pneumoniae* infection.

None of the uninfected (1 young, non-vaccinated, 2 young, vaccinated, and 3 aged, vaccinated) control mice had detectable *C. pneumoniae* titers (data not shown).

At 28 days p.i., 12 out of 13 (92%) young, non-vaccinated mice had *C. pneumoniae* lung titers (range: 1.0 x 10^2^- 5.0 x 10^3^ IFU/ml). The geometric mean titer for non-vaccinated young mice (5.4 x 10^2^ IFU/ml) was approximately 100-fold lower at 28 days p.i. than at 14 days p.i. (6.0 x 10^4^ IFU/ml), indicating that young C57BL/6 mice control respiratory *C. pneumoniae* infection to some degree without vaccination. Yet, clearance of the organism may be promoted by vaccination. At 28 days p.i., 9 of the 15 (60%) young, vaccinated mice had no evidence of *C. pneumoniae* in the lung (Figure 1B), compared to only 8% (1 of 13) in the non-vaccinated group. The remaining 6 (40%) young, vaccinated mice showed nominal infection (range = 5.0 x 10^2^-5.0 x 10^3^ IFU/ml, Figure 1B).

In contrast to that observed in young non-vaccinated mice, the infection established in aged mice following intranasal inoculation of *C. pneumoniae* appears to be progressive. The geometric mean titer from lungs of aged, unvaccinated mice at 28 days p.i. (1.9 x 10^4^ IFU/ml) was 10-fold higher than that obtained at 14 days p.i. (1.7 x 10^3^ IFU/ml). *C. pneumoniae* was detected in all (11/11) aged, non-vaccinated C57BL/6 mice at 28 days p.i. (Figure 1B). Still, the vaccine remains at least partially protective in aged mice; 5 of the 11 (45%) aged, vaccinated mice were clear of respiratory *C. pneumoniae* infection at 28 days p.i. The remaining 55% of aged, vaccinated mice had lung titers that ranged from 1.0 x 10^3^ to 2.0 x 10^4^ IFU/ml (Figure 1B). The geometric mean for all aged, vaccinated mice was 5.4 x 10^2^ IFU/ml which is 35-fold lower than that of aged, non-vaccinated animals, but not statistically significant (p = 0.109). These results suggest that the CTL response elicited in both young and aged mice still protects against respiratory infection in at least a subset of each group. Our results, however, indicate that a reservoir of infection existed over the 28 day period in some vaccinated mice, regardless of age. It is possible that the cytolytic response or the cytokines (including IFN-γ) produced by the CTL, drove the course of infection into a persistent state in some animals, as has been described by others [11].

No detectable *C. pneumoniae* titers were found in any uninfected mice (3 young non-vaccinated, 3 young vaccinated, 2 aged non-vaccinated and 5 aged vaccinated mice, data not shown).

## Effect of age and vaccination status on spread of *C. pneumoniae* to the cardiovascular system

**Figure 2:**
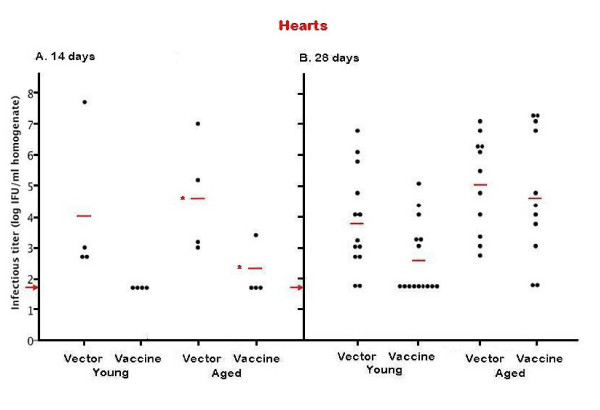
**Recovery of *C. pneumoniae *from the heart 14 and 28 days after intranasal inoculation of 5x10**^**5**^**IFU 6-month-old (young) or 20-month-old (aged) C57BL/6 mice were immunized with 100 μg of *Cpn*CTL7 (vaccine) or VR1012 plasmid DNA (vector) and challenged intranasally with 5x10**^**5**^**IFU*C. pneumoniae *12 days after completion of the immunization protocol.** Mice were euthanized 14 days (**A**) or 28 days (**B**) after infection, the hears/ascending aortae were removed, and lysates prepared as described in Materials and Methods. Viable organisms were recovered and quantified by immunofluorescent staining using FITC-conjugated *Chlamydia*-specific antibody (Fitzgerald 61-C75). The groups of experimentally infected and uninfected age-matched control mice are listed on the X-axis and the number of inclusion forming units/ml of 10 % weight/volume homogenate (log_10_) is displayed on the Y-axis. Each dot represents the concentration (IFU/ml) of *C. pneumoniae* recovered from the organ homogenate of an individual mouse. The red bars indicate the geometric mean of all animals in each group (log_10_) and the red arrow indicates the limit of C. pneumoniae in the detection system. The “*” symbol indicates a statistically significant difference (p = 0.001) between the geometric mean of respiratory titers of young, vaccinated mice and young mice receiving vector alone. The “**” symbols indicate a statistically significant difference (p = 0.012) between the geometric mean of respiratory titers of aged, vaccinated mice and aged mice receiving vector alone.

Cardiovascular infection is a common sequella of respiratory *C. pneumoniae* infection in both C57BL/6 (Figure 2) and BALB/c mice [6]. While cardiovascular infection may be established without overt symptoms, infection may trigger a chronic immune and/or inflammatory response that would contribute to the development and/or progression of pathology [6]. Thus, protective vaccination that would limit or prevent the spread of *C. pneumoniae* to the cardiovascular system might be an effective strategy in preventing or delaying age-related cardiovascular pathologies.

Our results show that, by 14 days p.i., all (4/4) of the young, non-vaccinated mice showed signs of *C. pneumoniae* spread to the heart/ascending aorta (geometric mean = 1.0 x 10^4^ IFU/ml, range: 5.0 x 10^2^- 5.0 x 10^7^ IFU/ml, Figure 2A). In contrast, none (0/4) of young vaccinated mice showed any signs of cardiovascular infection at 14 days p.i. (Figure 2A). Similarly, all 4 aged, non-vaccinated animals displayed signs of cardiovascular infection at 14 days p.i. (geometric mean = 3.9 x 10^4^ IFU/ml, range: 1.0 x 10^3^-1.0 x 10^7^ IFU/ml, Figure 2A). Only 1 of the 4 (25%) of the aged, vaccinated mice showed demonstrable cardiovascular infection (2.5 x 10^3^ IFU/ml) at 14 days p.i. (Figure 2A). The geometric mean for the aged, non-vaccinated mice was 288-times higher than that in aged, vaccinated mice (p = 0.025).

At 28 days p.i., 11 of the 13 (85%) young non-vaccinated mice had evidence of *C. pneumoniae* infection in the heart/ascending aorta (range: 5.0 x 10^2^-5.0 x 10^6^ IFU/ml, Figure 2B). Extra-respiratory spread was drastically reduced by vaccination in young mice. Only 40% (6 out of 15) of young vaccinated mice showed detectable infection in the heart/ascending aorta (range: 1.0 x 10^3^-1.0 x 10^5^ IFU/ml, Figure 2B). The geometric mean *C. pneumoniae* titer for the young, non-vaccinated animals (5.9x10^3^ IFU/ml) was 17-fold higher than that of young, vaccinated animals (3.4 x 10^2^ IFU/ml), a difference that was not statistically significant.

All (11 out of 11) of the aged, non-vaccinated animals displayed signs of infection in the heart/ascending aorta (range: 5.0 x 10^2^-1.0 x 10^7^ IFU/ml, geometric mean = 1.0 x 10^5^ IFU/ml, Figure 2B) at 28 days p.i. The vaccine, however, did not protect against extra-respiratory spread in aged animals; 9 of 11 (82%) of aged, vaccinated mice had detectable infections (range: 1.0 x 10^3^ to 1.5 x 10^7^ IFU/ml, Figure 2B) with a geometric mean of 4.5 x 10^4^ IFU/ml. These results indicate that while vaccination delays or prevents spread to the cardiovascular system in young mice, this strategy was not protective in aged mice; spread to the cardiovascular system was observed in 82% of aged, vaccinated mice by 28 days p.i.

These data suggest that, despite reports of intrinsic and extrinsic age-associated defects in CTL [12,13] including alterations in the diversity repertoire [reviewed in [14]], CD8+ CTL precursor, vaccine epitope-specific, cells must be induced in sufficient frequencies in aged mice to mount a protective response in the lung that closely parallels that seen in their young counterparts.

However, the CD8+ CTL response generated by the vaccine is not sufficient to provide long-term cardiovascular protection, especially in aged animals. One possibility is that extrinsic factors that could decrease the CTL response generated to the vaccine in aged mice may vary by site. Thus, the CTL response elicited by the vaccine, while remaining effective in the lungs, may have been inhibited in the cardiovascular system of aged mice via CD4^+^CD25^+^FoxP3^+^ regulatory cells, which increase in number during aging [15]. Another possible explanation of these findings is that the immune mechanisms that control respiratory *C. pneumoniae* burden differ from those that regulate extra-respiratory spread or the establishment of systemic infection. In this scenario, the CTL response generated by the vaccine may not prevent or may even promote spread to the cardiovascular system [11]. Finally, it is possible that non-immune age-related processes, such as atherosclerosis, contribute to the higher burden of cardiovascular infection and/or relative lack of vaccine efficacy in the aged. Wild type C57BL/6 mice fed normal low-fat laboratory chow, even at advanced age, do not show significant atherosclerotic changes within the aorta [16]. Still, other age-related changes inherent to or affecting the vascular endothelium may promote infection of these cells and thus, contribute to the burden of *C. pneumoniae* in the hearts/ascending aortae of aged mice.

## Abbreviations

CTL, Cytotoxic T lymphocyte; IFU, Infection forming units; HBSS, Hanks buffered salt solution; PBS, Phosphate buffered saline.

## Competing interests

The authors declare that they have no competing interests.

## Authors’ contributions

TE performed the immunofluorescence assays described herein, participated in the statistical analyses and critical analysis of the data, and wrote the first drafts of the manuscript. SB and AS performed the immunofluoresence assays. CSL and BB contributed significantly to the experimental design, performed the animal work, and provided critical analyses of the data, interpretations, and manuscript. DS prepared the vaccine and vector preparations for the study. BW, provided the vaccine and vector stocks for the study, contributed significantly to the experimental design, and provided critical analyses of the data, interpretations, and manuscript. KLF-D, (corresponding author) is the Principal Investigator in the laboratory and, as such, was centrally involved in all aspects of the work represented in this manuscript. All authors read and approved the final manuscript.

## References

[B1] Blasi F Tarsia P Aliberti S Cosentini R Allegra L Chlamydia pneumoniaeandMycoplasma pneumoniae Sem in Respiratory Critical Care Med 2005 26 617 624 10.1055/s-2005-925525 16388430

[B2] Campbell LA Kuo CC Chlamydia pneumoniae-an infectious risk factor for atherosclerosis Nature Rev Microbiol 2004 2 23 32 10.1038/nrmicro796 15035006

[B3] Mosorin M Surcel HM Laurila A Lehtinen M Karttunen R Juvonen J Paavonen J Morrison RP Saikku P Juvonen T Detection of Chlamydia pneumoniae-reactive T lympocytes in human atherosclerosis plaques of carotid artery Arterioscler Thromb Vasc Biol 2000 20 1061 1067 10.1161/01.ATV.20.4.1061 10764674

[B4] Gnarpe J Gnarpe H Gause-Nilsson I Lundorg P Steen B Seroprevalence of antibodies to Chlamydia pneumoniae in elderly people: a two-decade longitudinal and cohort study Scand J Infect Dis 2000 32 177 179 10.1080/003655400750045295 10826904

[B5] O’Neill C Murray LJ Ong GM O’Reilly DP Evans AE Bamford KB Epidemiology of Chlamydia pneumoniae infection in a randomly selected population in a developed country Epidemiol Infect 1999 122 111 116 10.1017/S0950268898001757 10098793 PMC2809595

[B6] Chen WH Kozlovsky BF Effros RB Grubeck-Loebenstein B Edelman R Sztein MB Vaccination in the elderly: an immunologic perspective Trends Immunol 2009 30 351 359 10.1016/j.it.2009.05.002 19540808 PMC3739436

[B7] Little CS Bowe A Lin R Litsky J Fogel RM Balin BJ Fresa-Dillon KL Age alterations in extent and severity of experimental intranasal infection with Chlamydophila pneumoniae in BALB/c mice Infect Immun 2005 73 1723 1734 10.1128/IAI.73.3.1723-1734.2005 15731073 PMC1064908

[B8] Vuola JM Puurula V Anttila M Makela PH Rautonen N Acquired immunity to Chlamydia pneumoniae is dependent on gamma interferon in two mouse strains that initially differ in this respect after primary challenge Infect Immun 2000 68 960 964 10.1128/IAI.68.2.960-964.2000 10639472 PMC97231

[B9] Rottenberg ME Gigliotti Rothfuchs AC Gigliotti D Svanholm C Bandholtz L Wigzell H Role of innate and adaptive immunity in the outcome of primary infection with Chlamydia pneumoniae, as analyzed in genetically modified mice J Immunol 1999 162 2829 2836 10072530

[B10] Pinchuk I Starcher BC Livingston B Tvninnereim A Wu S Appella E Sidney J Sette A Wizel B A CD8+ T Cell heptaepitope minigene vaccine induces protective immunity against Chlamydia pneumoniae J Immunol 2005 174 5729 5739 15843575 10.4049/jimmunol.174.9.5729

[B11] Pantoja LG Miller RD Ramirez JA Molestina RE Summersgill JT Characterization of Chlamydia pneumoniae persistence in HEp-2 cells treated with gamma interferon Infect Immun 2001 69 7927 7932 10.1128/IAI.69.12.7927-7932.2001 11705979 PMC98893

[B12] Effros RB Cai Z Linton PJ CD8 T cells and aging Crit Rev Immunol 2003 23 45 64 10.1615/CritRevImmunol.v23.i12.30 12906259

[B13] Iancu EM Speiser DE Rufer N Assessing ageing of individual T lymphocytes: Mission impossible Mech Ageing Devel 2008 129 67 78 10.1016/j.mad.2007.10.005 18048082

[B14] Yewdell JW Haeryfar SM Understanding presentation of viral antigens to CD8+ T cells in vivo: The key to rational vaccine design Ann Rev Immunol 2005 23 651 682 10.1146/annurev.immunol.23.021704.115702 15771583

[B15] Nishioka T Shimuzu J Iida R Yamazaki S Sagaguchi S CD4 + CD25 + FoxP3+ T cells in aged mice J Immunol 2005 176 6586 6593 16709816 10.4049/jimmunol.176.11.6586

[B16] Tennett GA Hutchinson WL Kahan MC Hirschfield GM Gallimore JR Lewin J Sabin CA Dhillon AP Pepys MB Transgenic human CRP is not pro-atherogenic, pro-atherothrombotic or pro-inflammatory in apoE−/− mice Atherosclerosis 2008 196 248 255 10.1016/j.atherosclerosis.2007.05.010 17588586

